# Eliminating cervical cancer from low- and middle-income countries: An achievable public health goal

**DOI:** 10.3389/fmed.2022.1096395

**Published:** 2022-12-20

**Authors:** Redhwan Ahmed Al-Naggar

**Affiliations:** ^1^Community Medicine Department, Faculty of Medicine, National University of Malaysia, Kuala Lumpur, Malaysia; ^2^Family Medicine Department, Faculty of Medicine, Aljeel Aljadeed University, Sana'a, Yemen

**Keywords:** cervical cancer, vaccination, low- and middle-income countries, elimination, prevention

It is possible to eliminate cervical cancer by fully vaccinating 90% of girls by the age of 15, screening 70% of women between the ages of 35 and 45, and treating 90% of cervical cancer patients in low- and middle-income countries. Cervical cancer can now be prevented with HPV vaccination and screening, as well as treated with effective surgery, radiotherapy, and chemotherapy. Nonetheless, over 500,000 new cases were reported worldwide (1).

Every year, half a million women worldwide are diagnosed with cervical cancer, with half of those diagnosed dying as a result. Cervical cancer is strongly linked to socioeconomic development, with the disease affecting low- and middle-income countries disproportionately (2).

Women in low and middle-income countries may not receive prophylactic vaccines or screening due to a lack of adequate logistics and infrastructure, as well as a lack of trained medical services and health education. Aside from political and social issues. It is critical to address these issues and devise strategies to overcome these obstacles. It is necessary to address the social, political, and economic barriers to accessing services, particularly those related to gender. Regardless of supply, cost, delivery, or skepticism, HPV vaccination should be made available to everyone who needs it.

Screening services must be established from scratch in some low-income countries. Screening positive women in countries where chemotherapy or radiotherapy are unavailable necessitate treatment. A large number of radiographers, physicists, nurses, and surgeons must be trained.

A multi-front battle will almost certainly be required to advance cervical cancer prevention. Raising public awareness about the problems caused by cervical cancer, as well as new discoveries about its etiology, is critical. Providing women and their male partners with updated health education appears to be a necessary first step.

Incorporating novel technology in a reasonable manner would reduce the number of visits required and make screening more efficient in terms of the protection provided by each visit. Educating professionals and populations about the vaccination strategy. Adopting an unbiased scientific approach to these issues is a critical responsibility of clinical and public health societies.

We are all capable of eliminating cervical cancer, and we are all aware of the health consequences of inaction. To overcome these challenges, governments, civil society, the private sector, and academia must now collaborate.

This Research Topic is an excellent starting point for the project. It includes nine articles from Nepal (Narasimhamurthy and Kafle), Brazil (Corrêa et al.), Philippines (Lintao et al.), Rwanda and Sierra Leone (Bangura et al.), China (Liu et al.) (Yu et al.), Peru (Shin et al.), Latin American countries (Rol et al.), and Kenya (Mabachi et al.), written by experts on various aspects of the field that describe the issues, options, and outcomes available.

The main focus of these studies from low and middle income countries is the importance of laboratory readiness in the successful implementation of HPV testing. High readiness, however, is insufficient to ensure high continuity capacity for HPV testing, which necessitates the establishment of a quality culture that includes regular training, robust monitoring, and quality assurance systems tailored to the local context. All efforts to improve HPV laboratories are valuable and necessary to ensure the successful implementation of HPV-based cervical screening (Rol et al.).

The incorporation of new technologies and approaches in those fronts is expected to help drive the country toward an elimination target, single-dose HPV vaccination, and HPV self-sampling testing, which are very effective cervical cancer prevention strategies in low- and middle-income countries. Local and international collaborations are also required to improve secondary prevention and management by establishing a reliable infrastructure to treat HPV-related pre-invasive and invasive cancer (Narasimhamurthy and Kafle and Corrêa et al.).

Other studies on the Research Agenda should be conducted, such as implementation research, epidemiological studies, and clinical research on the cost-effectiveness and efficacy of various traditional and novel treatment strategies, in order to develop a locally applicable system for implementing a national cervical cancer screening and HPV vaccination program (Lintao et al.). Improving data quality and completeness, which is a priority among national stakeholders, is critical. Investing in systems to improve the detection and treatment of precancerous lesions can improve patient outcomes and, in the long run, lower the costs associated with the much more expensive treatment of later-stage detection (Mabachi et al.).

Another issue raised by experts from low and middle income countries is awareness. Without policymakers' and the public's awareness and willingness to fund cervical cancer programs, the prioritization of cervical cancer activities, the availability of resources, an adequate health workforce and infrastructure, cross-sectional collaboration and planning, inter-sectorial, national, regional, and international partnerships, the goal of cervical cancer elimination would not be achievable in countries (Bangura et al.).

Another important issue raised by the experts is socioeconomic inequalities, risk factors, and community-based social entrepreneurship. In low and middle income countries, rural-urban inequality and socioeconomic inequalities continue to be problems (Liu et al.). Age, a history of alcohol consumption, marital status, reproductive diseases, education level, and the number of live births were all risk factors for HPV infection. Therefore, cervical cancer screening should be made available to women over the age of 30 in rural areas, particularly those aged 41–45 (Yu et al.). Future research is required to clarify the relationship between empowerment and worker performance in order to inform the expansion of HPV self-sampling social entrepreneurship programs (Shin et al.).

The completion of such a task at the appropriate time indicates that the international scientific community strongly supports low-income countries in the field of cervical cancer, and thus future scientific support should be available when required. Our challenge is to reduce the mortality rate from cervical cancer, an infectious disease with well-established and highly effective prevention methods. In this regard, the prospects for low and middle-income countries should be brighter than they have ever been.

## Author contributions

The author confirms being the sole contributor of this work and has approved it for publication.
